# Biannual Administrations of Azithromycin and the Gastrointestinal Microbiome of Malawian Children: A Nested Cohort Study Within a Randomized Controlled Trial

**DOI:** 10.3389/fpubh.2022.756318

**Published:** 2022-02-15

**Authors:** David Chaima, Harry Pickering, John D. Hart, Sarah E. Burr, Joanna Houghton, Kenneth Maleta, Khumbo Kalua, Robin L. Bailey, Martin J. Holland

**Affiliations:** ^1^Clinical Research Department, Faculty of Infectious and Tropical Diseases, London School of Hygiene and Tropical Medicine, London, United Kingdom; ^2^Microbiology Unit, Department of Pathology, College of Medicine, University of Malawi, Blantyre, Malawi; ^3^School of Public Health and Family Medicine, College of Medicine, University of Malawi, Blantyre, Malawi; ^4^Blantyre Institute of Community Outreach, Blantyre, Malawi; ^5^Department of Ophthalmology, College of Medicine, University of Malawi, Blantyre, Malawi

**Keywords:** mass drug administration, azithromycin, gut microbiota, V4-16S rRNA sequencing, amplicon

## Abstract

Community-level mass treatment with azithromycin has been associated with a mortality benefit in children. However, antibiotic exposures result in disruption of the gut microbiota and repeated exposures may reduce recovery of the gut flora. We conducted a nested cohort study within the framework of a randomized controlled trial to examine associations between mass drug administration (MDA) with azithromycin and the gut microbiota of rural Malawian children aged between 1 and 59 months. Fecal samples were collected from the children at baseline and 6 months after two or four biannual rounds of azithromycin treatment. DNA was extracted from fecal samples and V4-16S rRNA sequencing used to characterize the gut microbiota. *Firmicutes, Bacteroidetes, Proteobacteria* and *Actinobacteria* were the dominant phyla while *Faecalibacterium* and *Bifidobacterium* were the most prevalent genera. There were no associations between azithromycin treatment and changes in alpha diversity, however, four biannual rounds of treatment were associated with increased abundance of *Prevotella*. The lack of significant changes in gut microbiota after four biannual treatments supports the use of mass azithromycin treatment to reduce mortality in children living in low- and middle-income settings.

## Introduction

Azithromycin is a broad-spectrum, macrolide antibiotic characterized by a long intra- and extra-cellular half-life. Its use is indicated in the treatment of atypical pneumonia, skin and soft tissue infections and sexually transmitted infections (1). The World Health Organization (WHO) recommends mass azithromycin treatment at the community level as one of the key strategies for the elimination of trachoma as a public health problem (2). Studies of mass azithromycin distribution for trachoma control in endemic areas indicate that mass treatment has secondary effects, which include reductions in child morbidity and mortality (3–6). Recently, a multi-site trial, conducted in Malawi, Tanzania and Niger reported lower mortality rates in children (under 5 years of age) who received mass azithromycin treatment compared to children who received a placebo (7). The specific mechanism through which azithromycin reduced mortality in children is not understood, however, several studies have reported a reduction in the community burden of nasopharyngeal carriage of *Streptococcus pneumoniae* (8–10) and a reduction in the abundance of *Campylobacter* spp. in the gut (11). Additionally, reduced risks of diarrhea and acute lower respiratory infections related to azithromycin mass drug administration (MDA) for trachoma control have been reported in Tanzanian children (4, 5). Azithromycin may therefore reduce morbidity and mortality by reducing carriage of pathogenic bacteria.

Available evidence indicates that azithromycin treatment causes alterations in the gut microbiota that can be measured in the weeks immediately following treatment. Recent randomized, placebo-controlled trials of Parker et al. (12) and Wei et al. (13) characterized the intestinal microbiota of Indian and Danish infants respectively, at baseline and 14 days after a 3-day course of azithromycin and reported changes in intestinal microbiota. Both studies reported a decrease in alpha diversity in fecal samples of treated children compared to those who received placebo. Additionally, Parker et al. (12) reported a decrease in relative abundance of *Proteobacteria* and *Verrucomicrobia* while Wei et al. (13) reported a decrease in the abundance of *Actinobacteria* after treatment in fecal samples of treated children compared to placebo. Alterations in the gut microbiota have also been reported more than 6 months after exposure to antibiotics, suggesting that the short-term changes may persist for a longer period of time. An observational study by Korpela et al. (14), which characterized the fecal microbiota of Finnish children aged 2–7 years with varying durations of exposure to azithromycin, clarithromycin, penicillin or no antibiotic exposure over a 24-months span, showed a decrease in microbial richness in children treated with macrolides compared to those treated with penicillin or without exposure to antibiotics. Additionally, a significant decrease in the relative abundance of *Actinobacteria* and an increased abundance of *Bacteroidetes* and *Proteobacteria* was found in children who had used macrolide antibiotics within 6 months of sample donation. However, Wei et al. (13) reported no significant differences in alpha diversity and taxonomic composition between the treatment and control groups 4 years after treatment.

To date, it is not clear how long the azithromycin-related changes in the gut microbiota persist and whether such changes are augmented or amplified by repeated rounds of treatment. We conducted an exploratory study to investigate the association between azithromycin treatment and changes in the fecal microbiota of rural Malawi children 6 months after 2 or 4 biannual azithromycin distributions. We explored the diversity and composition of the intestinal microbiota in children who received azithromycin vs. children who received placebo.

## Materials and Methods

### Study Design

This study was nested within a survey of prevalence of carriage of macrolide resistant enteropathogens conducted within the Childhood Mortality Reduction after Oral Azithromycin in Malawi (MORDOR-Malawi) trials (NCT02048007, registration date 29 January 2014).

**Figure 1:**
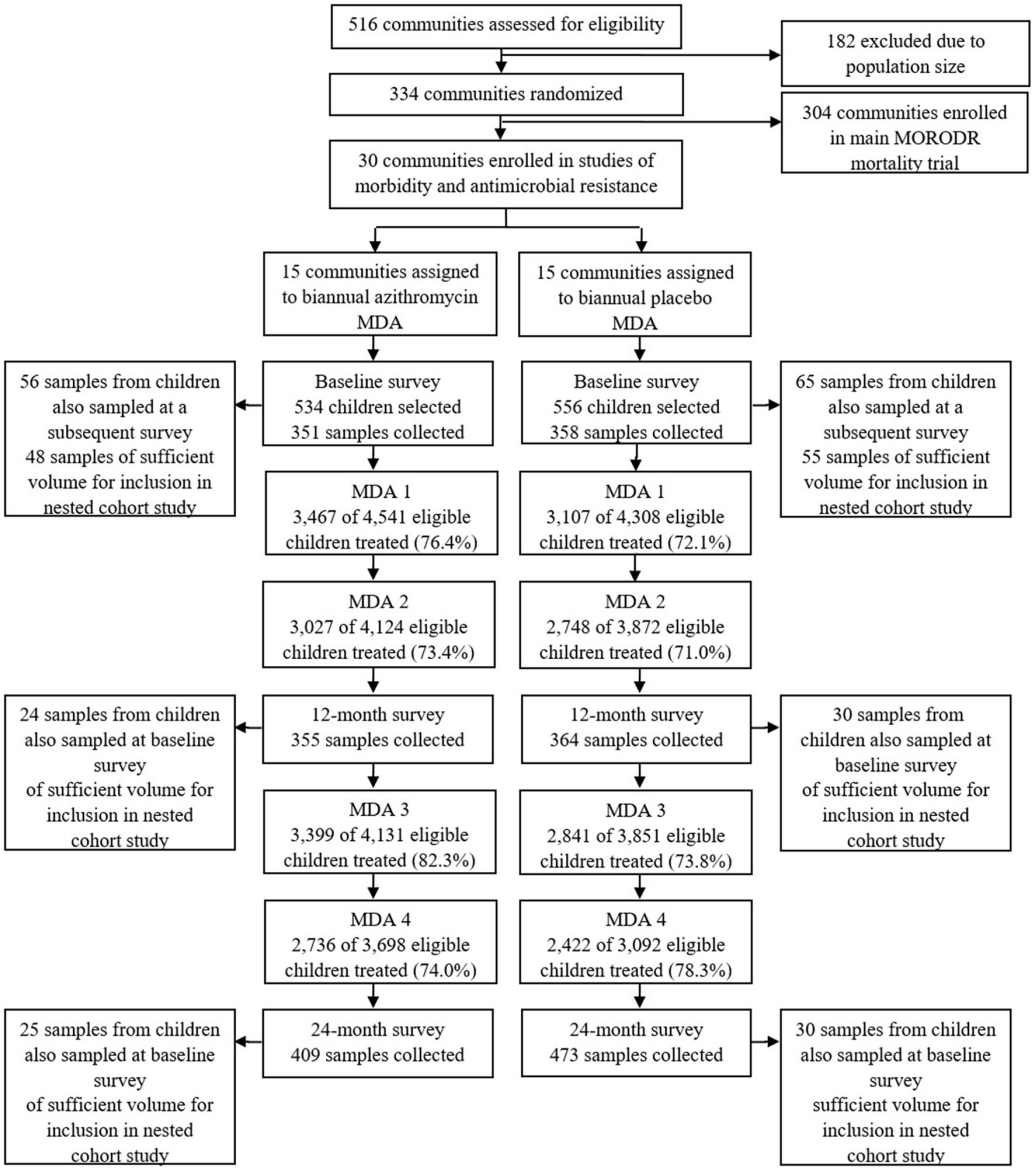
Study flow. Study flow for the cross sectional survey of carriage of antimicrobial resistance and selection of samples for the nested cohort study.

Briefly, in the MORDOR-Malawi study, 30 clusters in the Mangochi district, defined as the catchment area of a Health Surveillance Assistant (HSA) (~1,000 total population), were randomly selected to receive 4 biannual rounds of MDA with either azithromycin or placebo. All children aged 1–59 months and weighing ≥3.8kg were eligible for treatment at each of 4 biannual mass distributions. Fecal sample collection took place in the 30 clusters during the baseline survey (May-July 2015) and at 12-month and 24-month surveys (April-June 2016 and 2017, respectively), ~6 months after the second and fourth treatment rounds (Figure 1). At each survey, children aged 1–59 months were randomly selected for participation in sample collection using custom software on Android devices. The target for recruitment at each survey was 40 children in each of the 30 clusters, to recruit a total of 1,200 children. Azithromycin was administered at a dose of 20 mg/kg by HSAs and field-workers conducting house-to-house visits as detailed elsewhere (15).

A retrospective nested cohort study was then conducted within the framework provided by the three cross-sectional surveys described above. Of the recruited children who provided fecal samples at the baseline cross-sectional survey, 121 were also sampled, by chance, at an additional survey thereby providing the potential to generate longitudinal pairs of samples (before and after treatment). Baseline samples from 103 such children had sufficient volume remaining for 16S rRNA gene sequence analysis and were therefore included in the nested cohort study (Figure 1). Fifty-four of these children were sampled at the baseline and 12-month surveys and 55 children were sampled at the baseline and 24-month surveys (Figure 1).

### Outcome Measures

Stool samples were collected by the MORDOR-Malawi trial to determine the effect of treatment on the prevalence of carriage of macrolide resistance (pre-specified primary outcome), and on the diversity and composition of the intestinal microbiome (pre-specified secondary outcome measures). The nested cohort study described here did not form a part of the pre-specified analysis plan. It was conducted as an ancillary study after the trial had ended.

### Sample Size

The nested cohort study was an exploratory study that made use of available samples from the parent trial. No additional sample sizes were calculated.

### Fecal Sample Collection

Fecal samples from participating children were collected by their mothers or guardians, who were provided with sterile fecal collection bottles (Wheaton, UK) and given verbal instruction, by a nurse, on how to collect the sample. Fecal samples were returned to the field team as soon as possible after collection. These samples were put in a cool box with ice packs until the end of the day (not more than 8 h after collection) and were then brought to the laboratory where they were stored at −80°C.

### V4 16S rRNA Gene Sequencing

Samples were thawed at room temperature and 250 mg amounts weighed into sterile Eppendorf tubes. Total, genomic DNA was then extracted using the PowerSoil DNA Isolation Kit (MO BIO Laboratories Inc, Carlsbad, CA, now a part of Qiagen, Germany). Indexed primers 515F (515F_Indexed; 5'-adapter-GTGCCAGCMGCCGCGGTAA-3') and 806R (806R_Indexed; 5'-adapter-GGACTACHVGGGTWTCTAAT-3') (16) were used to amplify the V4 region of the bacterial 16S rRNA gene in the extracted DNA. The total volume of a single PCR reaction was 25 μl and each reaction contained 10 μl 2.5 × Quantabio 5prime HotMasterMix (Quantabio, Beverly, MA, USA), 10 μM primers (SIGMA, UK), 4 μl template DNA and 8 μl PCR grade water (Qiagen). Amplification took place on a Veriti™ 96 well-thermal cycler (Applied Biosystems, UK) using the following thermal cycling conditions: 94°C for 3 min, 35 cycles of 30 seconds at 94°C, 30 seconds at 50°C and 45 seconds at 72°C, then a final extension of 10 min at 72°C. Each PCR run included a mock bacterial community (composed of DNA from *Hemophilus influenza, Moraxella catarrhalis* and *Staphylococcus epidermis*), which acted as a positive control, and a no-template control. Amplicon size and PCR efficiency were verified by gel electrophoresis after which amplicons were purified using 0.6 v/v AMPure XP beads (Beckman Coulter, CA, USA) and 70% ethanol.

A second PCR was then performed to barcode each amplicon thereby enabling downstream multiplexing. The total volume for a single reaction of index PCR was 49 μl. Each 49 μl PCR reaction contained 20 μl 2.5 × Quantabio 5prime HotMasterMix (Quantabio, Beverly, MA, USA), 2 μM MID Illumina primers (17), 22 μl of amplicon and 5.5 μl of PCR grade water (Qiagen). Cycling conditions were as follows: 94°C for 3 min followed by 5 cycles of 10 s at 94°C, 30 s at 58°C and 45 s at 72°C, then 10 min at 72°C.

Amplicons were purified, quantified on a Qubit 2.0 Fluorometer (Invitrogen, Carlsbad, CA, USA) and thereafter pooled in equimolar amounts. The MiSeq v3 reagent kit (Illumina Inc., San Diego, CA, USA) was used to prepare the DNA library for cluster generation and sequencing. The DNA library was denatured with a sodium hydroxide solution (0.2N NaOH) and then diluted with hybridization buffer (HT1) to a final loading concentration of 15 pM. It was then spiked with 10% PhiX control, which served as an internal control for low-diversity libraries, heat-denatured at 96°C for 2 min and assayed by 2×300 bp paired-end sequencing on the Illumina MiSeq platform for a total of 600 cycles using a standard protocol (18).

### Sequence Processing

FastQ files, containing raw sequence data, were processed in QIIME 2 software (version 2018.6) (19). Barcoded sequences were demultiplexed using the *demux* function in QIIME 2. Poor quality reads were filtered out and chimeras were removed.

The 16S rRNA sequences were clustered *de novo* into OTUs at ≥ 97% identity. Taxonomy was assigned to the OTUs using a naïve Bayes classifier pre-trained on the SILVA 16S database (20). To exclude spurious OTUs, only bacterial OTUs identified to the genus level, with sequences more than 0.005% of the total number of sequences (21) and a frequency of more than 0.01% in any sample were retained in the analyses.

### Statistical Analysis

All statistical analyses were performed in R (22). Shannon (H) and Simpson (D) diversity indices were calculated using the phyloseq package (23). Differences in the distribution of parametric data between groups were tested using Student's *t*-test or ANOVA while the Wilcoxon-rank or Kruskal-Wallis tests were used for non-parametric data. A linear regression model with the participant as a random effect was used to examine whether change in microbiota diversity within individuals over time was different in those receiving azithromycin or placebo. The sample proportion test was used to compare proportions between groups. To determine bacterial phylogenetic distance between samples, weighted and unweighted UniFrac distance matrices were calculated using the phyloseq R package. The phylogenies used to calculate the UniFrac distances were computed using RAxML v8.2.11147 from a variable sites alignment using a generalized time-reversible (GTR) + gamma model. To compare bacterial community compositional differences between groups, PERMANOVA with 1000 permutation tests was run on weighted and unweighted UniFrac distances and principal coordinate analysis (PCoA) plots were used to visualize differences in UniFrac clustering by groups. Generalized linear mixed models using the participant as a random effect were used to examine changes in individual genera between time points.

### Ethical Considerations

The MORDOR-Malawi study was approved by the London School of Hygiene and Tropical Medicine ethics committee in the UK (study number 6500) and the College of Medicine Research Ethics Committee in Malawi (study number P.02/14/1521). Information and consent forms were translated into local languages (Yao and Chichewa) prior to their approval by the local ethics committee. Consent was first obtained at the community level through discussions with the village chief and community elders who then indicated the willingness, or unwillingness, of the community to participate through verbal consent. Written, informed consent (by thumbprint or signature) was then obtained from the parent or guardian of each child before recruitment, including consent for their child's samples to be sent to the UK for testing that was not available in Malawi. Parents and guardians informed of their freedom to withdraw their child from the study at any time without giving reason for doing so. All subjects gave their informed consent for inclusion before they participated in the study. The study was conducted in accordance with the Declaration of Helsinki.

## Results

### Baseline Demographics

A baseline survey of prevalence of carriage of macrolide resistant enteropathogens enrolled 1,090 children, however, of these, only 709 (65%) returned fecal samples. Of these 709 children, 103 (9%) were included in a nested cohort study that examined the fecal microbiota using 16S rRNA sequence analyses. One baseline sample consistently failed to amplify and could not be sequenced; 102 samples were retained for analysis after quality filtering. The age and sex of children whose fecal samples were included in the analysis of the nested cohort compared to all children enrolled in the cross-sectional survey are shown in Table 1. The proportion of male children was comparable between those whose fecal samples were included in this nested study and all enrolled participants. However, children whose fecal samples were included in the nested cohort were younger compared to all enrolled participants.

**Table 1:** Baseline demographics of participants whose samples were included in the nested cohort study of 16S rRNA sequencing vs. all participants who provided fecal samples for the cross sectional survey of antimicrobial resistance.

**Participant characteristic**	**Children included in nested cohort**	**All enrolled children**	***P*-value**
Number of participants (*n*)	102	1,090	
Male sex N (%)	52 (51%)	487 (45%)	0.304^¥^
Mean (SD) age in months	22.95 (13.54)	29.59 (16.42)	<0.001^*^
Age range in months	2–56	1–59	

¥ *Denotes P-values obtained from sample proportion test while*

* *denotes P-values obtained from Student's t-test*.

### Number of Sequencing Reads, Characteristics and Taxon Abundance at Baseline

**Figure 2:**
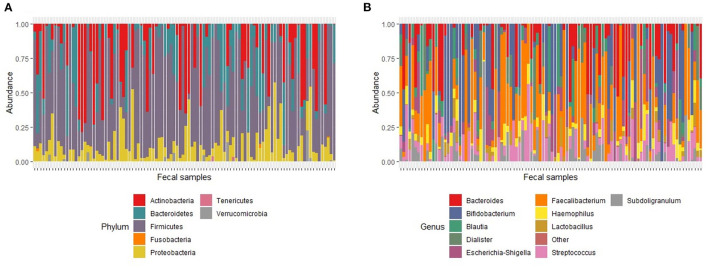
Relative abundance of major taxa found in fecal samples at baseline. **(A)**. Major phyla. The proportion of total number of reads for each phylum in a sample represents phylum abundance after rarefaction to 1000 reads. **(B)** Major genera. The proportion of total number of reads for each genus in a sample represents genus abundance after rarefaction to 1000 reads. The stacked bar plot only shows the 10 most abundant genera. Genera with relative abundance <1% were grouped as “Other.”

One hundred and two baseline samples were retained for analysis after quality filtering. The baseline dataset generated a total of 1,685,792 reads with an average read depth per sample of 16,527 reads (range; 4182–69401). A total of 9 phyla, 16 classes, 25 orders, 43 families and 117 classified and 6 unclassified genera were identified from the V4-amplicon sequence reads. Seven genera (*Faecalibacterium, Streptococcus, Bacteroides, Haemophilus, Subdoligranulum, Bifidobacterium* and *Escherichia-Shigella*) were present in approximately 60% of the samples and together accounted for 46% of the whole bacterial community. After rarefaction to 1000 reads with 1000 permutations, 69 genera belonging to 7 phyla, *Actinobacteria, Bacteroidetes, Firmicutes, Fusobacteria, Proteobacteria, Tenericutes* and *Verrucomicrobia* were retained. Out of the 7 identified phyla, *Firmicutes, Bacteroidetes, Proteobacteria* and *Actinobacteria* were the most dominant and accounted for 64%, 14%, 9% and 7% of the total bacterial community, respectively (Figure 2A). Seven genera (*Faecalibacterium, Streptococcus, Bacteroides, Haemophilus, Subdoligranulum, Bifidobacterium* and *Escherichia-Shigella*) were present in ~62% of the samples and together accounted for 46% of the whole bacterial community; *Faecalibacterium* was prevalent in 81% of the samples making it the most prevalent genus. *Bifidobacterium* was prevalent in 51% of the samples and had the highest number of reads, accounting for 15% of the total (Figure 2B).

### Definition of Datasets and Baseline Demographics of the Datasets

Of the 102 baseline samples included in the analysis, 54 came from children who were also sampled at the 12-month time point while 55 came from children who were also sampled at the 24-month time point. Nine children were sampled at all three time points (baseline, 12 and 24 months).

Samples from 54 children who were sampled at baseline and at the 12-month survey, which was conducted 6 months after 2 rounds of biannual treatment, were included in the final analysis. The data from these samples are referred to as the “BL vs 2MDA dataset.” Samples from 55 children who participated in baseline sampling and sampling at the 24-month survey, conducted 6 months after 4 rounds of biannual treatment, were also included in the final analysis. This dataset is referred to as the “BL vs 4MDA dataset.”

Within the BL vs. 2MDA dataset, 30 children received placebo and 24 received azithromycin treatment while within the BL vs. 4MDA dataset, 30 children received placebo and 25 received azithromycin treatment (Figure 1). Age and sex of children were comparable between azithromycin and placebo arms in both data sets (Table 2).

**Table 2:** Comparison of baseline participant characteristics between treatment groups for the BL vs. 2MDA and BL vs. 4MDA datasets.

	**Treatment arm**		***P*-value**
**Participant characteristics**	**Azithromycin**	**Placebo**	
**BL vs. 2MDA dataset**			
Number of participants (*n*)	24	30	
Child male sex *N* (%)	14 (58%)	15 (50%)	0.74^¥^
Mean (SD) age, months	27 (14)	24.3 (15)	0.51^*^
**Treatment rounds received** ***n*** **(%)**			
0	1 (4%)		
1	10 (42%)		
2	13 (51%)		
**BL vs. 4MDA dataset**			
Number of participants (*n*)	25	30	
Child male sex, *n* (%)	13 (52%)	16 (53%)	0.99^¥^
Mean (SD) age, months	18.3 (10.6)	19 (10.8)	0.82^*^
**Treatment rounds received** ***n*** **(%)**			
0	3 (12)		
1	3 (12)		
2	6 (24)		
3	4 (16)		
4	9 (36)		

¥ *Denotes P-values obtained from sample proportion test while*

* *denotes P-values obtained from Student's t-test*.

Of the 30 communities that were part of the main trial, 27 (90%) are represented in the present analysis. Of the 27 communities, 14 were randomized to azithromycin whilst the remaining 13 were randomized to placebo.

### Alpha Diversity Measures of the Datasets at Baseline

We examined the distribution of alpha diversity indicated by Shannon and Simpson indices in the BL vs. 2MDA and BL vs. 4MDA datasets. Within the BL vs. 2MDA dataset, Shannon and Simpson indices were different between azithromycin and placebo arms at baseline, however, both diversity indices were similar between the two treatment groups at baseline within the BL vs. 4MDA dataset (Table 3).

**Table 3:** Alpha diversity distribution between treatment groups.

**Dataset**	**Time-point**	**Shannon diversity index**			**Simpson diversity index**		
		**Azithromycin**	**Placebo**	***P*-value**	**Azithromycin**	**Placebo**	***P*-value**
BL vs. 2MDA (*n* = 54)	Baseline	2.4 (1.9, 2.5)^¥^	2 (1.6, 2.2)^¥^	0.02	0.9 (0.8, 0.9)^¥^	0.8 (0.7, 0.9)^¥^	0.01
	12 months	2.2 (2, 2.4)^¥^	2.2 (1.7, 2.4)^¥^	0.71^a^	0.8 (0.8, 0.9)^¥^	0.8 (0.7, 0.8)^¥^	0.72^a^
BL vs. 4MDA (*n* = 55)	Baseline	2.0 (0.5)^α^	2.1 (0.5)^α^	0.69^b^	0.8 (0.7, 0.8)^¥^	0.8 (0.7, 0.8)^¥^	0.61
	24 months	2.2 (0.5)^α^	2.2 (0.4)^α^	0.97^b^	0.8 (0.8, 0.9)^¥^	0.8 (0.8, 0.9)^¥^	0.91^a^

¥ *Median (25^th^, 75^th^ quartile)*.

α *Mean (SD)*.

a *P-value calculated by Wilcoxon rank sum test*.

b *P-value calculated by Student's t-test*.

### No Change in Alpha Diversity Measures Observed Following 2 and 4 Treatment Rounds

The BL vs. 2MDA dataset was used to explore changes in microbiota diversity 6 months after 2 rounds of azithromycin MDA between and within individuals. At the 12-month survey, no differences in alpha diversity, measured by Shannon and Simpson diversity indices, were found between the azithromycin and placebo treatment arms (Table 3). Similarly, there was no difference in Shannon or Simpson diversity index distribution between baseline and 12-month fecal samples within the azithromycin arm (Table 4). To examine whether change in microbiota diversity within individuals over time was different in those receiving azithromycin or placebo, we performed a linear regression of alpha diversity on time of sampling in each study arm, with the participant as a random effect. This intra-individual analysis showed no change in Shannon or Simpson diversity in the azithromycin arm, there was similarly no change in the placebo arm (Supplementary Table 1).

**Table 4:** Alpha diversity distribution within the azithromycin treatment group.

**Dataset**	**Shannon index**				**Simpson index**		
**BL vs. 2MDA**	**Baseline**	**12 months**	***P*-value**		**Baseline**	**12 months**	***P*-value**
	2.1 (0.5) ^¥^	2.2 (0.5) ^¥^	0.88^a^		0.8 (0.11) ^¥^	0.8 (0.11) ^¥^	0.99^a^
**BL vs. 4MDA**	**Baseline**	**24 months**	***P*** **-value**	***P*** **-value**	**Baseline**	**24 months**	***P*** **-value**
	1.8 (0.5)^α^	2.2 (0.5)^α^	0.005^b^	0.37^c^	0.77 (0.17)^¥^	0.82 (0.10)^¥^	0.33^a^

¥ *Median (25^th^,75^th^ quartile)*.

α *Mean (SD)*.

a *P-value calculated by Wilcoxon rank sum test*.

b *P-value calculated by Student's t-test*.

c *Denotes P-values calculated from a linear regression model after adjusting for age and sex*.

The BL vs. 4MDA dataset was used to assess the effect of up to 4 rounds of biannual azithromycin treatment on fecal microbiota diversity 6 months after the last treatment round between and within individuals. A comparison of fecal microbiota diversity between azithromycin and placebo arms after 4 rounds of azithromycin had been distributed showed no differences in Shannon and Simpson diversity indices (Table 3). A comparison of Shannon and Simpson diversity indices between fecal samples collected baseline and 24-month surveys in the azithromycin group revealed differences between the two time-points however these were not found after adjusting for age and sex (Table 4). As described above for the BL vs. 2MDA comparison, intra-individual analysis showed no change in Shannon or Simpson diversity in the azithromycin arm, there was similarly no change in the placebo arm (Supplementary Table 1).

### Small Scale Changes in Microbiota Composition Following 2 and 4 Treatment Rounds

Within the BL vs. 2MDA dataset, PERMANOVA analysis using unweighted (R^2^ = 0.01, F = 0.69, *P* = 0.85) and weighted (R^2^ = 0.01, F = 0.47, *P* = 0.89) UniFrac distances did not detect differences in overall microbiota composition between azithromycin and placebo treatment arms following two rounds of treatment. Similarly, there were no differences in microbiota composition between the fecal samples collected at the baseline and 12-month surveys in the azithromycin group based on weighted (R^2^ = 0.03, F = 1.39, *P* = 0.21) and unweighted (R^2^ = 0.03, F = 1.35, *P* = 0.14) UniFrac distances.

Within the BL vs. 4MDA dataset, PERMANOVA analysis did not detect any differences in overall fecal microbiota composition between azithromycin and placebo groups after four treatment rounds, based on weighted (R^2^ = 0.005, F = 0.24, *P* = 0.97) and unweighted (R^2^ = 0.015, F = 0.79, *P* = 0.73) UniFrac distances.

**Figure 3:**
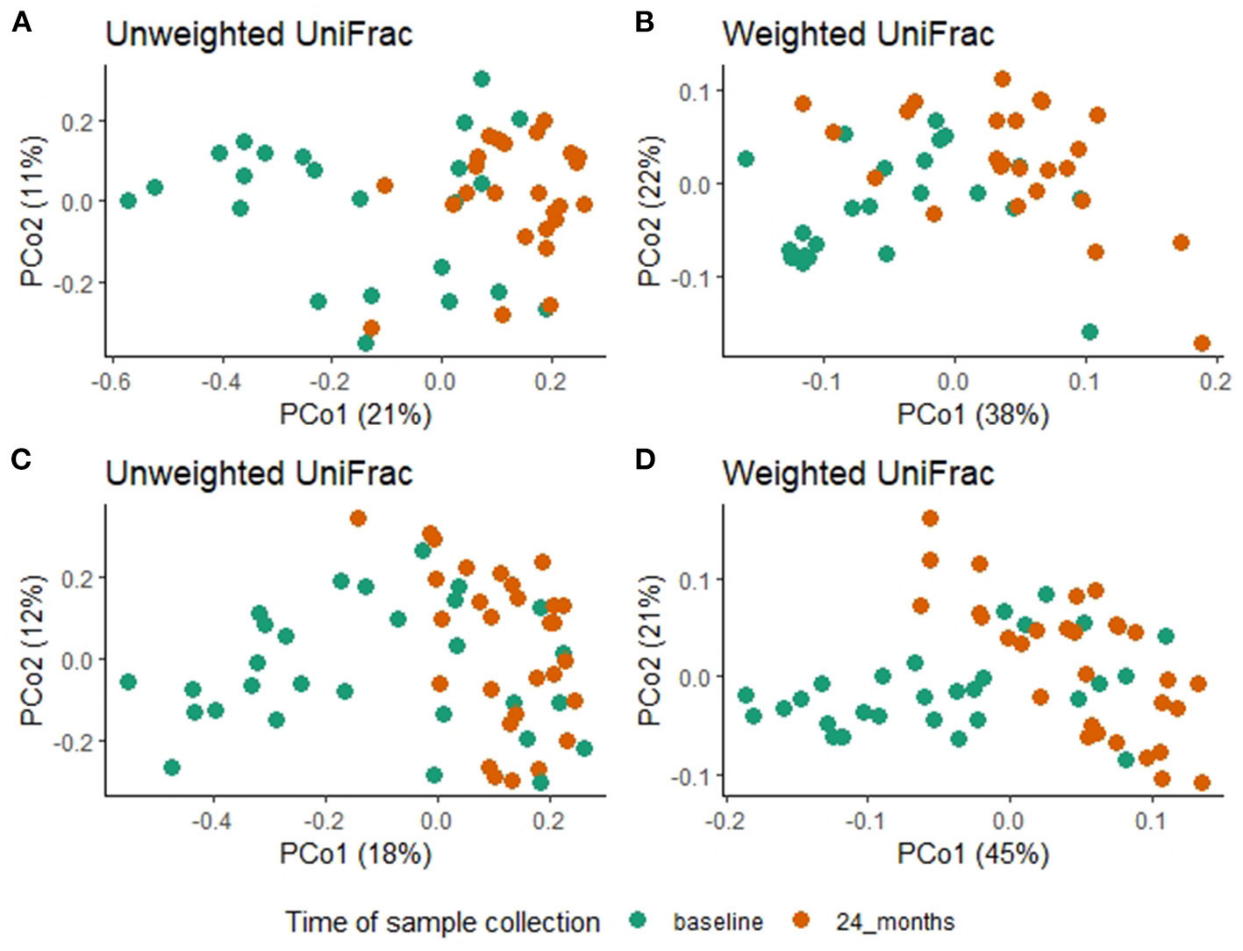
Overall fecal microbiota composition differences between baseline and 24-month fecal samples in the azithromycin **(A,B)** and placebo **(C,D)** arms. **(A)** PCoA plot of unweighted UniFrac distances [PERMANOVA, R^2^ = 0.1, F = 5.09, *P* = 0.001] **(B)** PCoA plot of weighted UniFrac distances [PERMANOVA, R^2^ = 0.1, F = 5.1, *P* = 0.001], **(C)** PCoA plot of unweighted UniFrac distances [PERMANOVA, R^2^ = 0.07, F = 4.01, *P* = 0.001] **(D)** PCoA plot of weighted UniFrac distances [PERMANOVA, R^2^ =0.16, F=11.87, P=0.001]. In all the PCoA plots above **(A–D)**, PCo1 explained the most variation and baseline fecal samples clustered in the negative space of PCo1 while 24-month fecal samples clustered in the positive space of PCo1.

**Figure 4:**
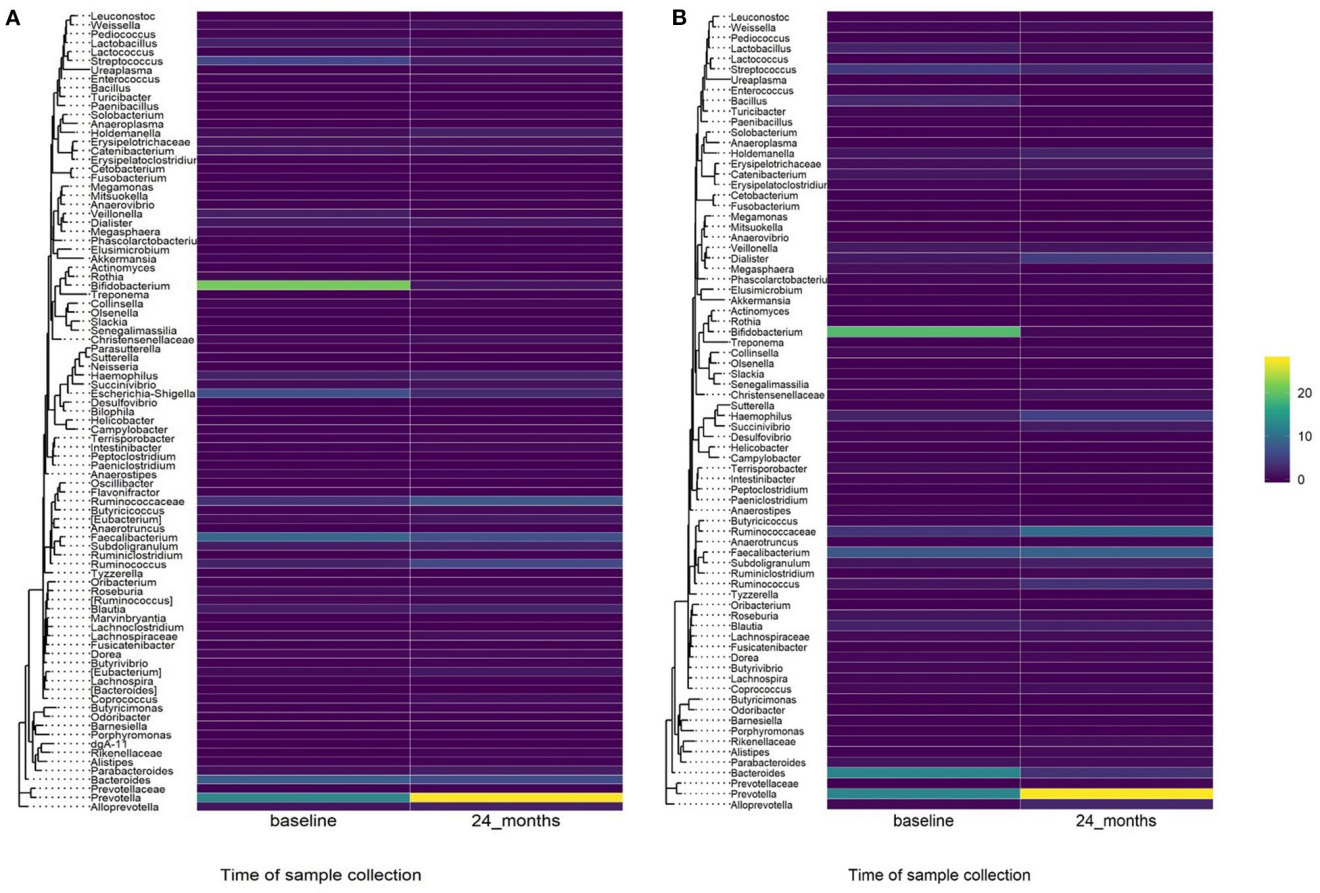
Heatmap showing relative abundance of genera in baseline and 24-month fecal samples by treatment arm. **(A)** Azithromycin arm, **(B)** Placebo arm.

A comparison of fecal microbiota composition between baseline and 24-month within the azithromycin arm, after distribution of 4 rounds of azithromycin, showed significant differences based on unweighted (Figure 3A) and weighted (Figure 3B) UniFrac distances. The relative abundances of taxa at baseline and after 4 rounds of treatment within the azithromycin arm were compared and visualized using stacked bar plots at phylum and genus levels. Crude comparisons of relative abundance at the phylum level showed a higher relative abundance of *Bacteroidetes* after treatment [median (IQR) of 0.38 (0.31, 0.45) at the 24-month survey vs. 0.21 (0.08, 0.40) at the baseline survey, Wilcoxon *P* = 0.004]. *Actinobacteria* was shown to be more abundant in fecal samples collected at baseline in the azithromycin arm [median (IQR) of 0.08 (0, 0.47) at baseline vs. 0.01 (0, 0.02) at the 24-month survey, Wilcoxon *P* = 0.014]. At the genus level, *Bifidobacterium* and *Streptococcus* were more abundant in samples collected at baseline compared to the 24-month survey samples, while *Prevotella* was more abundant in fecal samples collected at the 24-month survey (Table 5 and Figure 4A).

**Table 5:** Longitudinal comparison of relative abundance of individual genera after 4 rounds of biannual treatment compared to baseline in the azithromycin and placebo arms within the BL vs. 4MDA dataset.

**Treatment arm**	**Genera**	**Coefficient (95% CI)^a^**	***P*-value^a^**	**Coefficient (95% CI)^b^**	***P*-value^b^**
Azithromycin	*Bifidobacterium*	−3.9 (−5.9, −1.9)	<0.01	0.18 (−0.79, 1.16)	0.71
	*Prevotella*	1.5 (0.6, 2.4)	0.002	0.3 (0.003, 0.6)	0.05
	*Streptococcus*	−40 (−70, −9)	0.012	0.54 (−0.1, 1.17)	0.1
Placebo	*Bifidobacterium*	−3.3 (−4.7, −1.8)	<0.01	0.66 (−0.68, 2.0)	0.34
	*Prevotella*	1.3 (0.6, 1.9)	0.0002	0.21 (−0.28, 0.69)	0.4

*CI, confidence interval*.

a *Coefficient (95% CI) and P-values obtained with unadjusted linear regression model*.

b *Coefficient (95%CI) and P-values obtained with linear mixed models adjusted for age, sex and time since last treatment with the participant as a random effect*.

To test whether the crude differences observed between the baseline and 24-month surveys in the azithromycin group could be due to treatment or natural changes in the microbiota, we then compared the baseline and 24-month samples within the placebo arm. A comparison of microbiota composition between baseline and 24-month surveys within the placebo group also showed significant differences based on unweighted (Figure 3C) and weighted (Figure 3D) UniFrac distances. Relative abundance of the phylum *Actinobacteria* was shown to be higher in samples collected at baseline than at 24-month [median (IQR) of 0.14 (0.00–0.31) at the baseline survey vs. 0.0001 (0.00–0.01) at the 24-month survey, Wilcoxon *P* = 0.001]. At the genus level, *Bifidobacterium* was shown to be more abundant in fecal samples collected at baseline compared to 24-month samples while *Prevotella* was more abundant in samples collected at the 24-month survey (Table 5 and Figure 4B).

To further examine changes in genera that were differentially abundant at the group-level, we tested for individual-level changes and their relationship to receiving either azithromycin or placebo. For this, we performed a linear regression of genera abundance on time of sampling in the azithromycin and placebo arms separately, with the participant as a random effect. The analyses showed no significant differences in the relative abundance of *Streptococcus* between the baseline and 24-month surveys in the azithromycin arm. There were also no significant differences in the relative abundance of *Bifidobacterium* between the baseline and 24-month surveys in both the azithromycin and placebo arms. However, azithromycin treatment was associated with increased relative abundance of *Prevotella* at the 24-month survey; samples collected at the 24-month survey in the azithromycin arm had a 0.3 increase in the relative abundance of *Prevotella* compared to samples collected at the baseline survey within the same azithromycin arm (Table 5). In contrast, no longitudinal differences were shown in the placebo arm in the adjusted analysis (Table 5).

## Discussion

We examined associations between 2 and 4 biannual azithromycin treatment rounds and changes in intestinal microbiota diversity and composition in children resident in treated communities. Azithromycin administration was not associated with changes in alpha diversity but was weakly associated with changes in gut microbiota composition after 4 biannual treatment rounds.

The lack of an effect of azithromycin treatment on microbiota diversity is consistent with data by Wei et al. (13), although the follow-up period in the present study was shorter. In the study by Wei et al. (13), Danish children aged 12–36 months were prescribed a 3-day course of azithromycin or placebo and the gut microbiota was characterized in fecal samples collected from each child at 4 years of follow-up. The study reported no significant differences in alpha diversity (measured by observed richness and Shannon index) between the treatment and placebo groups.

Conflicting results on alpha diversity have been recently reported by the MORDOR trial in Niger (24). A reduction in inverse Simpson and Shannon diversity indices in children who received azithromycin compared to children who received placebo was detected 6 months after 2 biannual treatment rounds. The discrepancy in the findings between that study and our own could be attributed to differences in sequencing methods. In the present study, we performed V4-16S rRNA sequencing while the study in Niger performed whole genome sequencing, which has higher resolution to detect differences.

Four biannual azithromycin treatment rounds in the present study were weakly associated with increased abundance of *Prevotella*, detectable 6 months after the last treatment round. While this finding has not previously been reported, it is consistent with a recent study that reported an increased abundance of *Bacteroidetes*, the phylum to which *Prevotella* belongs, in children who received macrolides within the 6 months preceding sample collection (14). *Prevotella* is a gram-negative commensal bacterium found at mucosal sites of the respiratory tract, gut, and oral cavity. Reduced abundance of this bacterium has been associated with Crohn's disease in pediatric patients (25). Therefore, a trend toward increased abundance of *Prevotella* 6 months after 4 biannual azithromycin treatment rounds may suggest potential long-term beneficial effects of mass azithromycin treatment to the gut, although further studies from additional study sites would be needed to validate this finding.

Our observation that there were small scale changes in gut microbiota composition 6 months after 4 biannual rounds of azithromycin treatment but not 6 months after 2 biannual rounds suggests that the long-term effects of azithromycin MDA on the gut microbiota may be dependent on the number of treatment rounds. The absence of large-scale changes in the gut microbiota 6 months after 2 or 4 biannual azithromycin treatment rounds suggests that any effects of azithromycin mass treatment on the gut microbiota may last days or weeks after treatment but not months. Furthermore, the absence of large scale changes in the gut microbiota may be regarded as a positive finding considering that large scale changes in the gut microbiota lead to gut microbiota imbalance, which increases the host's susceptibility to pathogens that can cause a variety of diseases including diarrhea (26).

Our study has several limitations. The study was reliant on samples collected in larger cross-sectional surveys and participation in those surveys was low. At the baseline survey, 1,090 children were enrolled and provided with stool sample collection kits. However, only 709 (65%) returned stool samples. Similar participation rates were seen at the 12- and 24-month surveys. This may have resulted in selection bias, particularly if families of children who participated had different health-seeking behaviors than those families of children who chose not to participate. Furthermore, we did not calculate sample size needed for this exploratory study as the samples included in the present nested cohort analyses were selected based on the availability of longitudinal pairs (baseline and 12 months or baseline and 24-month). Aside from the possibility of selection bias, the number of available longitudinal samples may not have had enough power to detect differences between treatment arms. Another limitation was that not all of the participants who were sampled received antibiotic as scheduled at each treatment round. While it is most unusual to achieve 100% treatment coverage in MDA programs, having participants miss treatment might have contributed to the magnitude of the effect of azithromycin on the gut microbiota reported by this study.

## Data Availability Statement

The datasets presented in this study can be found in online repositories. The names of the repository/repositories and accession number(s) can be found at: https://www.ebi.ac.uk/ena, PRJEB41238.

## Ethics Statement

The studies involving human participants were reviewed and approved by College of Medicine Research Ethics Committee and London School of Hygiene and Tropical Medicine Ethics Committee. Written informed consent to participate in this study was provided by the participants' legal guardian/next of kin.

## Author Contributions

SB, KK, RB, and MH: conceptualization, resources, and funding acquisition. DC, HP, JHo, and JHa: methodology. DC and HP: formal analysis. DC, JHa, and KK: data curation. DC: writing – original draft preparation. DC, HP, SB, KM, KK, RB, and MH: writing – review & editing. JHo, SB, KK, RB, and MH: supervision. KK and RB: project administration. All authors read and approved the final manuscript.

## Funding

Funding for this work was provided by the Bill and Melinda Gates Foundation, under grant numbers OPP1066930 and OP1032340. The funder of the study played no role in the study design and the preparation of the manuscript.

## Conflict of Interest

The authors declare that the research was conducted in the absence of any commercial or financial relationships that could be construed as a potential conflict of interest.

## Publisher's Note

All claims expressed in this article are solely those of the authors and do not necessarily represent those of their affiliated organizations, or those of the publisher, the editors and the reviewers. Any product that may be evaluated in this article, or claim that may be made by its manufacturer, is not guaranteed or endorsed by the publisher.

## References

[1] McMullan B Mostaghim M. Prescribing azithromycin. Aust Prescr. (2015) 38:87–9. 10.18773/austprescr.2015.030 26648627 PMC4653965

[2] Emerson PM Burton M Solomon AW Bailey R Mabey D. The SAFE strategy for trachoma control: Using operational research for policy, planning and implementation. Bull World Health Organ. (2006) 84:613–9. 10.2471/BLT.05.28696 16917648 PMC2627433

[3] Porco TC Gebre T Ayele B House J Keenan J Zhou Z. Effect of mass distribution of azithromycin for trachoma control on overall mortality in Ethiopian children: a randomized trial. JAMA. (2009) 302:962–8. 10.1001/jama.2009.1266 19724043

[4] Coles CL Seidman JC Levens J Mkocha H Munoz B West S. Association of mass treatment with azithromycin in trachoma-endemic communities with short-term reduced risk of diarrhea in young children. Am J Trop Med Hyg. (2011) 85:691–6. 10.4269/ajtmh.2011.11-0046 21976574 PMC3183779

[5] Coles CL Levens J Seidman JC Mkocha H Munoz B West S. Mass distribution of azithromycin for trachoma control is associated with short-term reduction in risk of acute lower respiratory infection in young children. Pediatr Infect Dis J. (2012) 31:341–6. 10.1097/INF.0b013e31824155c9 22173140

[6] Whitty CJ Glasgow KW Sadiq ST Mabey DC Bailey R. Impact of community-based mass treatment for trachoma with oral azithromycin on general morbidity in Gambian children. Pediatr Infect Dis J. (1999) 18:955–8. 10.1097/00006454-199911000-00003 10571428

[7] Keenan JD Bailey RL West SK Arzika AM Hart J Weaver J. Azithromycin to reduce childhood mortality in Sub-Saharan Africa. N Engl J Med. (2018) 378:1583–92. 10.1056/NEJMoa1715474 29694816 PMC5849140

[8] Leach AJ Shelby-James TM Mayo M Gratten M Laming AC Currie BJ. A prospective study of the impact of community-based azithromycin treatment of trachoma on carriage and resistance of Streptococcus pneumoniae. Clin Infect Dis. (1997) 24:356–62. 10.1093/clinids/24.3.356 9114185

[9] Burr SE Milne S Jafali J Bojang E Rajasekhar M Hart J. Mass administration of azithromycin and Streptococcus pneumoniae carriage: cross-sectional surveys in the Gambia. Bull World Health Organ. (2014) 92:490–8. 10.2471/BLT.13.133462 25110374 PMC4121870

[10] Adegbola RA Mulholland EK Bailey R Secka O Sadiq T Glasgow K. Effect of azithromycin on pharyngeal microflora. Pediatr Infect Dis J. (1995) 14:335–7. 10.1097/00006454-199504000-00024 7603826

[11] Doan T Hinterwirth A Worden L Arzika AM Maliki R Abdou A. Gut microbiome alteration in MORDOR I: a community-randomized trial of mass azithromycin distribution. Nat Med. (2019) 25:1370–6. 10.1038/s41591-019-0533-0 31406349

[12] Parker EPK Praharaj I John J Kaliappan SP Kampmann B Kang G. Changes in the intestinal microbiota following the administration of azithromycin in a randomised placebo-controlled trial among infants in south India. Sci Rep. (2017) 7:9168. 10.1038/s41598-017-06862-0 28835659 PMC5569098

[13] Wei S Mortensen MS Stokholm J Brejnrod AD Thorsen J Rasmussen MA. Short- and long-term impacts of azithromycin treatment on the gut microbiota in children: a double-blind, randomized, placebo-controlled trial. EBioMedicine. (2018) 38:265–72. 10.1016/j.ebiom.2018.11.035 30478001 PMC6306380

[14] Korpela K Salonen A Virta LJ Kekkonen RA Forslund K Bork P. Intestinal microbiome is related to lifetime antibiotic use in Finnish pre-school children. Nat Commun. (2016) 7:10410. 10.1038/ncomms10410 26811868 PMC4737757

[15] Hart JD Samikwa L Sikina F Kalua K Keenan JD Lietman TM. Effects of biannual azithromycin mass drug administration on malaria in malawian children: a cluster-randomized trial. Am J Trop Med Hyg. (2020) 103:1329–34. 10.4269/ajtmh.19-0619 32342841 PMC7470590

[16] Caporaso JG Lauber CL Walters WA Berg-Lyons D Lozupone CA Turnbaugh PJ. Global patterns of 16S rRNA diversity at a depth of millions of sequences per sample. Proc Natl Acad Sci U.S.A. (2011) 108(Suppl. 1):4516–22. 10.1073/pnas.1000080107 20534432 PMC3063599

[17] van Overbeek M Capurso D Carter Matthew M Thompson Matthew S. DNA repair profiling reveals nonrandom outcomes at cas9-mediated breaks. Mol Cell. (2016) 63:633–46. 10.1016/j.molcel.2016.06.037 27499295

[18] Illumina. Miseq System Guide. (2013). Available online at: https://support.illumina.com/sequencing/sequencing_instruments/miseq/documentation.html (accessed June, 2018).

[19] Bolyen E Rideout JR Dillon MR Bokulich NA Abnet C Al-Ghalith GA. QIIME 2: Reproducible, interactive, scalable, and extensible microbiome data science. PeerJ Preprints. (2018) 6:e27295v27292. 10.7287/peerj.preprints.27295v1 31341288 PMC7015180

[20] Yarza P Yilmaz P Pruesse E Glöckner FO Ludwig W Schleifer KH. Uniting the classification of cultured and uncultured bacteria and archaea using 16S rRNA gene sequences. Nat Rev Microbiol. (2014) 12:635. 10.1038/nrmicro3330 25118885

[21] Bokulich NA Subramanian S Faith JJ Gevers D Gordon JI Knight R. Quality-filtering vastly improves diversity estimates from Illumina amplicon sequencing. Nat Methods. (2013) 10:57–9. 10.1038/nmeth.2276 23202435 PMC3531572

[22] R Core Team (2020). R: A Language and Environment for Statistical Computing. Vienna: R Foundation for Statistical Computing. Available online at: https://www.R-project.org/

[23] McMurdie PJ Holmes S. Phyloseq: an R Package for reproducible interactive analysis and graphics of microbiome census data. PLoS ONE. (2013) 8:e61217. 10.1371/journal.pone.0061217 23630581 PMC3632530

[24] Doan T Hinterwirth A Arzika AM Cotter SY Ray KJ O'Brien KS. Mass azithromycin distribution and community microbiome: a cluster-randomized trial. Open Forum Infect Dis. (2018) 5:ofy182. 10.1093/ofid/ofy182 30151409 PMC6101535

[25] Lewis JD Chen EZ Baldassano RN Otley AR Griffiths AM Lee D. Inflammation, antibiotics, and diet as environmental stressors of the gut microbiome in pediatric crohn's disease. Cell Host Microbe. (2015) 18:489–500. 10.1016/j.chom.2015.09.008 26468751 PMC4633303

[26] Gresse R Chaucheyras-Durand F Fleury MA Van de Wiele T Forano E Blanquet-Diot S. Gut microbiota dysbiosis in postweaning piglets: understanding the keys to health. Trends Microbiol. (2017) 25:851–73. 10.1016/j.tim.2017.05.004 28602521

