# Supplementary material for: Biannual Administrations of Azithromycin and the Gastrointestinal Microbiome of Malawian Children: A Nested Cohort Study Within a Randomized Controlled Trial
Source: Front Public Health. 2022 Feb 15;10:756318. doi: 10.3389/fpubh.2022.756318 (PMC8885630; doi:10.3389/fpubh.2022.756318)
Supplement: Supplementary file 1 [file Table_1.docx]

**Supplementary table**

**Supplementary table 1:** Intra-individual variation in microbiota diversity

| **Dataset** | **Diversity index** | **Azithromycin** | | | **Placebo** | | |
| --- | --- | --- | --- | --- | --- | --- | --- |
|  |  | Estimate | Std.Error | p value | Estimate | Std.Error | p value |
| **BL vs 2MDA** | Shannon | -0.1 | 0.11 | 0.387 | 0.01 | 0.13 | 0.965 |
|  | Simpson | -0.03 | 0.02 | 0.168 | 0.01 | 0.03 | 0.788 |
| **BL vs 4MDA** | Shannon | 0.15 | 0.2 | 0.458 | -0.02 | 0.18 | 0.925 |
|  | Simpson | -0.01 | 0.05 | 0.875 | -0.01 | 0.04 | 0.817 |
